# Cardiovascular Risk Assessment of Elevated Blood Pressure for Screening and Early Detection in Children 6 to 9 Years of Age in the Valencian Community: ANIVA Study

**DOI:** 10.3390/children10121928

**Published:** 2023-12-14

**Authors:** Maria Morales-Suarez-Varela, Isabel Peraita-Costa, Agustin Llopis-Morales, Jorge Navarro Perez

**Affiliations:** 1 Research Group in Social and Nutritional Epidemiology, Pharmacoepidemiology and Public Health, Department of Preventive Medicine and Public Health, Food Sciences, Toxicology and Forensic Medicine, Faculty of Pharmacy, Universitat de València, Av. Vicent Andrés Estelles s/n, 46100 Burjassot, Spain; 2 Biomedical Research Center in Epidemiology and Public Health Network (CIBERESP), Carlos III Health Institute, Av. Monforte de Lemos 3-5 Pabellón 11 Planta 0, 28029 Madrid, Spain; 3 Foundation for Research of the Clinical Hospital of the Valencian Community (INCLIVA), C. de Menéndez y Pelayo 4, 46010 Valencia, Spain; 4 Department of Medicine, Faculty of Medicine, Universitat de València, Av. de Blasco Ibáñez 15, 46010 Valencia, Spain

**Keywords:** children, cardiovascular, screening, blood pressure

## Abstract

Elevated blood pressure (EBP), hypertension (HT), and prehypertension (PHT), along with the rising prevalence of overweight/obesity in children, correlate with a heightened risk of cardiovascular complications. This study focuses on assessing the prevalence of overweight/obesity and EBP and identifying potential indicators for effective early screening and detection of EBP in children aged 6 to 9 years old. This cross-sectional study was conducted with 1142 students from different schools across the Valencian Community in Spain. Data collection involved administering a questionnaire alongside direct anthropometric measurements of each student. The collected data underwent comprehensive statistical analysis, including frequencies, percentages, means, and chi-square automatic interaction detector (CHAID) analysis. In the sample, 7.5% of the children had HT and 6.8% had PHT, meaning 14.3% presented with EBP. Additionally, 17.0% of the children were classified as overweight and 3.9% as obese. Body mass index >23.1, body fat percentage >20.79%, and Kidmed scores <8 were identified as potential markers for early detection of EBP. The study reveals a significant incidence of EBP and overweight/obesity. Implementing screening protocols for early detection of EBP is imperative to forestall future cardiovascular events. Moreover, lifestyle modifications emerge as the most crucial approach for managing these risk factors.

## 1. Introduction

Cardiovascular disease (CVD) is a pressing global public health concern, with a well-established link between cardiovascular risk factors (CVRFs) and cardiovascular events (CVEs) [1]. Notably, arterial hypertension (HT) significantly contributes to cardiovascular morbidity [2]. Among modifiable CVRFs, lifestyle-related factors originating in childhood can persist into adulthood, serving as early indicators of adult CVRFs and subsequent CVDs [3,4]. At birth, most children have ideal cardiovascular health, yet lifestyle behaviors often lead to exposure to modifiable CVRFs during their lives [5,6]. While at birth, children without congenital CV abnormalities or pathologies have ideal CV health, most will be affected by modifiable CVRFs at some point in their lives due to lifestyle-related behaviors [7].

Primary HT is increasingly prevalent in children, paralleling the global rise in obesity [8]. A comprehensive study spanning 25 years (1990–2014) across five continents reported a global pooled prevalence of pediatric HT of 4.00% (95% CI, 3.29–4.78%), with increasing prevalence over the reported period [9]. Other important findings were a 9.67% (95% CI, 7.26–12.38%) prevalence of prehypertension (PHT), a 4.00% (95% CI, 2.10–6.48%) prevalence for stage 1 HT, and a 0.95% (95% CI, 0.48–1.57%) prevalence for stage 2 HT in children [10]. Meanwhile, a Spanish study noted a prevalence increase from 6.6% to 10.6% when transitioning from the 2016 European Society of Hypertension guidelines to the 2017 American Academy of Pediatrics guidelines [11]. Despite the acknowledged significance of HT, definitions and guidelines for pediatric HT vary [10], highlighting the impact of changing standards on prevalence data and underscoring the need for focused identification, assessment, and management of associated risk factors, particularly lifestyle modifications [8,12].

Childhood obesity (body mass index (BMI) ≥ 95th percentile) and overweight (BMI between 85th and 94th percentile) are known CVRFs [13] that are becoming increasingly widespread. Over the last half century, the estimated worldwide prevalence of obesity has increased to around 6% in boys and 8% in girls [14]. While obesity and overweight are widely considered independent CVRFs, they are also linked to other resultant CVRFs, such as elevated blood pressure (EBP), defined as either PHT or HT [15,16]. In children with obesity or overweight, the risk of having EBP is >3 times higher when compared to normal-weight children [17].

Sustaining lifestyle changes started in adulthood is difficult; therefore, it is recommended that healthy lifestyle behaviors are implemented as early as possible, and major improvements could be made by following public health strategies targeting the modifiable behaviors related to these CVRFs. The early detection of children with CVRFs and their treatment using targeted lifestyle modifications may aid in decreasing the prevalence of childhood CVRFs and possibly reduce the burden of later CV.

This study aims to ascertain the prevalence of EBP in children aged 6–9 years in the Valencian Community, Spain. It seeks to understand the weight and BP status of local schoolchildren, recognizing that EBP is often the first sign of CV risk. Additionally, the study aims to identify indicators for screening, early detection, intervention, and/or treatment of PHT and HT to mitigate CV risk.

## 2. Methods

This study complies with the guidelines of the Declaration of Helsinki developed by the World Medical Association. Prior to conducting this study, institutional permissions were obtained from the Autonomous Secretariat of Education and the University of Valencia (approval of the Ethics Committee: 2014/29630).

Informed consent forms and information sheets were provided to the parents or legal guardians of the students, describing the objectives and methodology of the study and inviting them to participate. Parents/legal guardians were asked to sign the informed consent form and return it to the research team if they were willing to allow their children to be included in the study. The project was then explained to all the children whose parents agreed to take part in the study.

The initial sample was made up of all children, six to nine years old, of both sexes, attending the participating schools. Children who did not correctly complete the take-home questionnaires or were absent when the anthropometric and CV measurements were taken were excluded. The rate of participation in this study, within the project “Anthropometry and Infant Nutrition of Valencia” (ANIVA), was 60.82%. The final sample consisted of 1142 children, belonging to primary schools in the province of Valencia.

All anthropometric and CV determinations were carried out in a private space with adequate supervision, thus guaranteeing conditions of intimacy and a relaxed atmosphere for participating children. To minimize interobserver variability, measurements were taken in standardized conditions, with anthropometric measurements taken following World Health Organization (WHO) standard procedures [18,19] and BP measurements following the protocols of the Spanish Association of Pediatrics (AEP) [20].

Weight, height, and body fat percentage measurements were taken while the children were barefoot and wearing light clothing. Weight measurements were taken using a Seca^®^ 861 scale (Vogel and Halke, Hamburg, Germany), and height was measured with a Seca^®^ 222 wall stadiometer (Vogel and Halke, Hamburg, Germany). Measurements were taken twice, and the mean was recorded. Body mass index (BMI, kg/m^2^) was calculated using these mean measurements. Body fat percentage was measured using a Tanita^®^ Segmental-418 bioimpedance analysis system (Tanita Corp., Tokyo, Japan). Two readings were made under controlled conditions: the children were barefoot, in a fasted state, had urinated, and had been sitting down and resting for 15 min.

The measurement of the skin folds was made using a Holtain Ltd.^®^ (Crymych, UK) caliper (0.2 mm precision and constant pressure of 10 g/mm^2^ between the valves). Three measurements were taken, and the mean was recorded. The triceps skin fold measurement was taken in the upper posterior region of the arm, at the midway point between the bottom of the olecranon process and the bony protrusion of the shoulder. The biceps skin fold measurement was made at the midpoint of the biceps brachii at the ventral region level. The suprailiac skin fold was measured in the mid-axillary line, just above the iliac crest. For the abdominal skin fold measurement and using the midpoint of the navel as the initial reference point, a horizontal skin fold 3 cm to the right and 1 cm below was used.

A flexible and inextensible tape measure for measurement of perimeters + BMI (Quirumed S.L.^®^ (Paterna, Spain), model: SKBMI-64) was used to measure waist circumference. The measurement was taken halfway between the tenth rib and the iliac crest after a normal breath. The same tape measure was used to measure hip circumference. This measurement was taken on the right side at the point of maximum circumference on the buttocks, placing the tape in a horizontal plane with the ground, and after a normal breath. Three measurements were taken, and the mean was recorded. Both of these mean measurements were used to obtain the waist–hip ratio.

Three separate measurements, five minutes apart, were taken for heart rate (HR) and BP (systolic (SBP) and diastolic (DBP)) using an automatic tensiometer (Omron-M5-I, Omron Healthcare Europe BV^®^, Hoofddorp, The Netherlands) and the most appropriate cuff size. Before the first measurement, the child must have been resting for a minimum of five minutes. Measurements were taken in a quiet and relaxed environment. The child was seated, and their right arm was positioned semi-flexed at heart level in accordance with the National High Blood Pressure Education Program Working Group on High Blood Pressure in Children and Adolescent recommendations [21,22]. The mean of the three measurements was recorded. Mean BP (MBP) and pulse pressure (PP) were calculated as follows: MBP = DBP + (0.333 × [SBP − DBP]) and PP = SBP − DBP.

Following the guidelines of the National High Blood Pressure Education Program Working Group on High Blood Pressure in Children and Adolescent [21,22], BP categorization was determined according to the sex, age, and height-dependent BP percentiles. If average SBP or DBP levels are ≥90th percentile but <95th percentile, a child is considered to have PHT, and HT if average SBP and/or DBP is ≥95th percentile.

To assess nutritional status, a questionnaire and food journal were given to the parents. In the questionnaire, they were asked about their child’s food allergies or intolerances, the use of prescribed and over-the-counter medications, the use of mineral and vitamin supplements, and physical activity. In the food journal, they were asked to write down all the food and drinks ingested by their child for three days, two school days, and one holiday [23,24]. They were asked to write down the ingredients in detail and serving sizes and quantities, as well as the brands of food and drinks consumed. Parents were given instructions in order to be able to correctly estimate food and drink intake.

Children were classified as underweight, normal weight, overweight, or obese according to BMI values calculated using the Anthro Plus^®^ computer software (https://www.who.int/tools/growth-reference-data-for-5to19-years/application-tools accessed on 15 May 2023) and the sex and age-dependent percentile values for a Spanish pediatric population (Supplementary Table S1). Body fat percentage, waist circumference, hip circumference, waist-hip ratio, triceps skin fold, biceps skin fold, suprailiac skin fold, and abdominal skin fold were categorized as low (first quartile), medium (second and third quartile), or high (fourth quartile).

The normality of the distribution of continuous variables was verified using both graphic (probability diagram) and statistical (Kolmogorov-Smirnov test) methods. All the variables were found to have a normal distribution; therefore, the parametric test was used in the analysis. Anthropometric variables and BP values were presented as mean and standard deviation. The quantitative variables were analyzed using a Student’s t-test. The Chi-square test (with Bonferroni correction) was used to compare the differences between variables. The data was analyzed using the IBM SPSS 26 Statistics software, and the criterion of statistical significance was established at *p* ≤ 0.05.

In addition, Chi-square Automatic Interaction Detector (CHAID) analysis was performed to determine the effect of independent variables on BP values. CHAID analysis is a decision tree technique based on adjusted significance testing used to discover the relationship between a categorical response variable and other categorical predictor variables, created by Gordon V. Kass in 1980 [25]. CHAID analysis is useful to detect patterns in datasets with a large number of variables as it builds a predictive model to help determine the independent variable interactions that best explain the chosen dependent variable outcome.

The first step of the CHAID analysis is the identification of the dependent variable. CHAID analysis separates the dependent variable into two or more categories, and then these categories are further separated using statistical algorithms. The analysis evaluates all possible interactions until no further separation can be done, meaning the best outcome has been reached.

Some of the advantages of CHAID analysis in comparison to other analytical methods are that all data types (nominal, ordinal, and continuous) can be used, a normal distribution of the data is not required, and it provides a simpler presentation of the relationship between the variables that can be easily visualized.

## 3. Results

The children participating in the study had a mean age (years) of 7.21 ± 1.00; 47.5% were male and 52.5% were female. The personal anthropometric and cardiovascular characteristics of the sample are shown in Table 1.

The results obtained identified some significant differences in anthropometric indicators and BP by sex. Boys had higher weight, height for age z-score, weight for age z-score, and BMI for age z-score, while girls had a higher SBP and HR. Significant differences were not found in the prevalence of the BP categories between the sexes.

**Figure 1:**
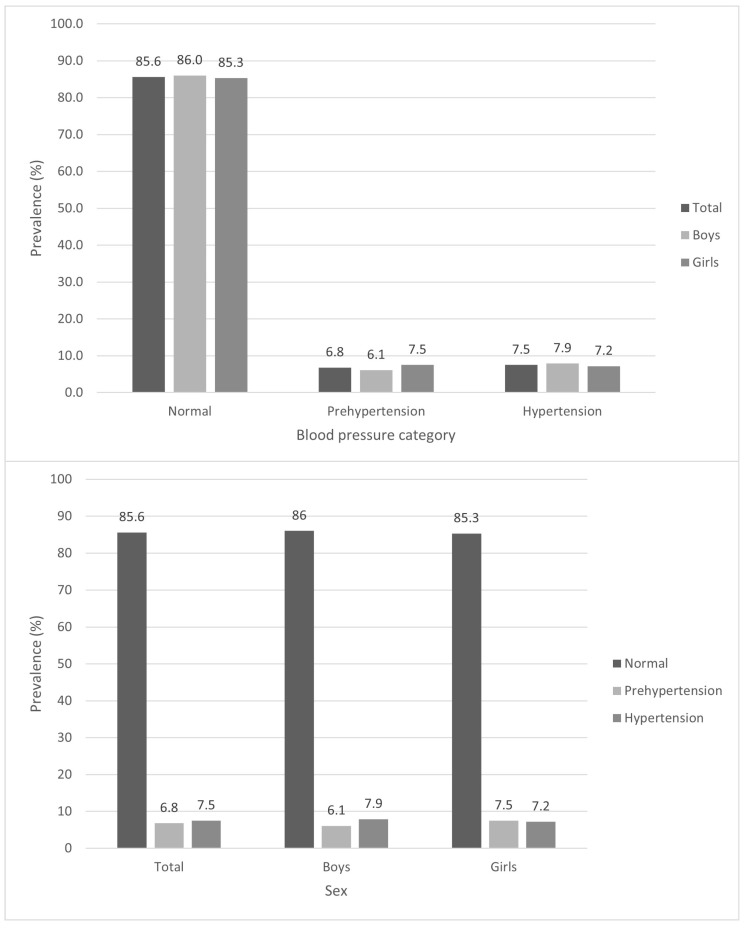
Prevalence of blood pressure categories by sex.

Figure 1 shows the prevalence of the BP categories by sex. The results obtained confirm a higher prevalence of normal BP, with 85.6%, without significant difference between sexes, and it is boys who present a greater percentage of normal BP, with 86.0%. Estimates of PHT and HT were 6.8% and 7.5%, respectively, with no significant difference between boys and girls. Girls present a greater PHT, with 7.5%, compared to boys, who present 6.1%. Boys present a higher percentage with HT, with 7.9% compared to 7.2% of the girls. No statistical differences were found between the estimated prevalences of PHT and HT by sex groups.

**Figure 2:**
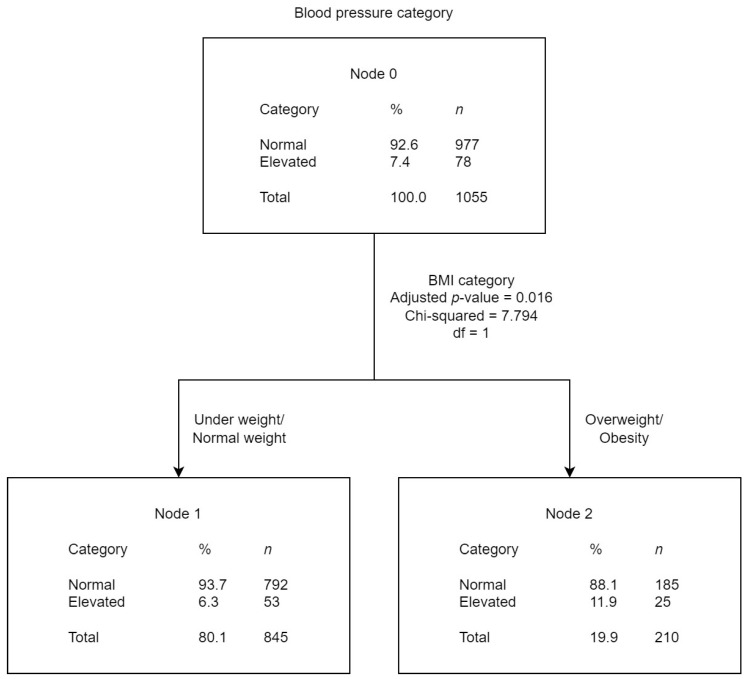
Blood pressure category CHAID analysis with BMI category as the independent variable.

**Figure 3:**
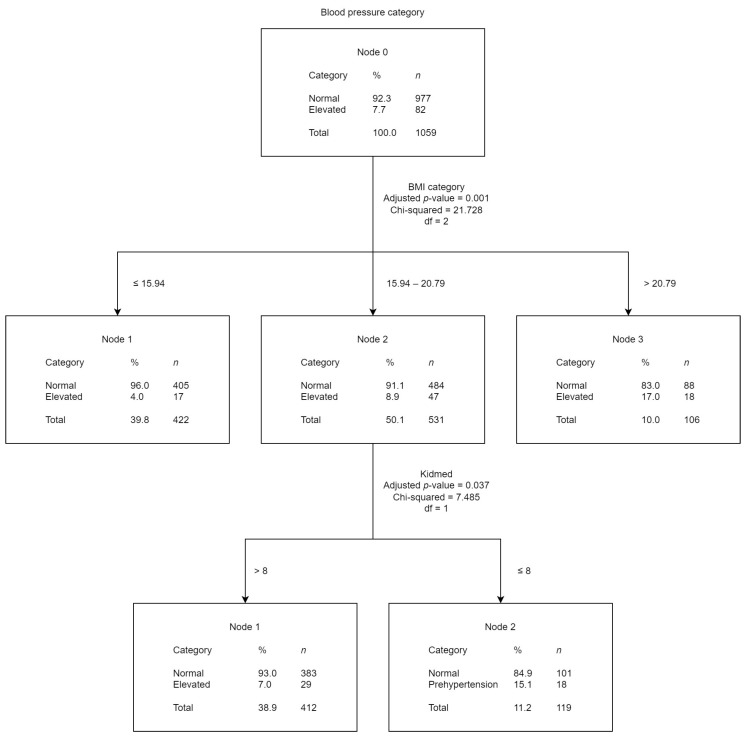
Blood pressure category CHAID analysis with BMI and Kidmed score as the independent variables.

**Figure 4:**
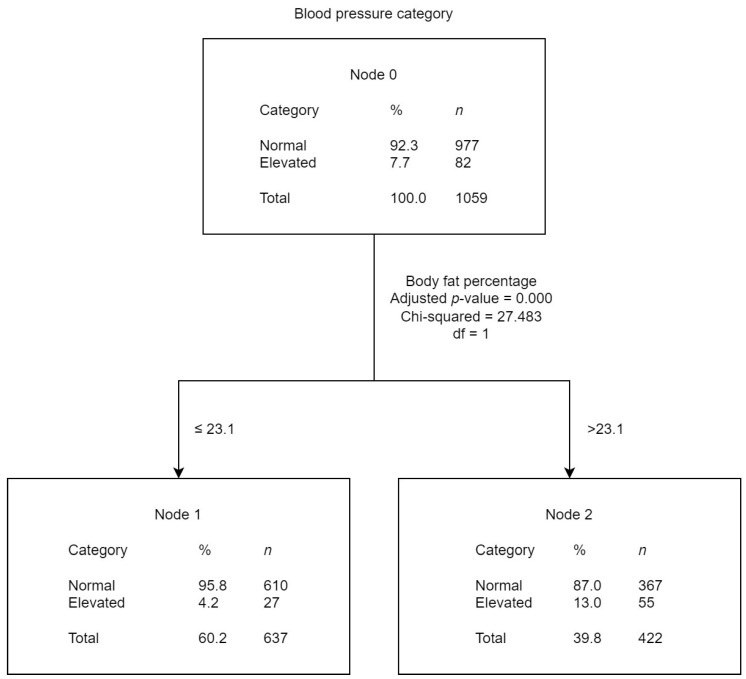
Blood pressure category CHAID analysis with body fat percentage as the independent variable.

The tree diagrams (Figure 2, Figure 3 and Figure 4) show the results obtained from the CHAID analysis, where the BP category (normal and PHT) was the dependent variable and other risk factors were the independent variables. The diagrams show that 92.3% of the children included in the analysis had normal BP and 7.7% EBP. The figures present the results of the CHAID analysis of the studied possible risk factors that best accounted for the BP categorization.

The CHAID analysis showed that (Figure 2), “BMI category” was the independent variable that best explained BP categorization (*p* = 0.016). The classification accuracy for this model was 95.1% (*p* = 0.008), meaning that with the divisions established by the model for the independent variable (“BMI category”), individuals would be classified in their correct BP category 95/100 times. Accordingly, the percentage of EBP was found to be higher in students within the obese BMI category.

The second tree diagram results (Figure 3) showed that “BMI” was the independent variable that best explained BP categorization (*p* = 0.001). In the secondary-level nodes of the diagram, “Kidmed score” (*p* = 0.037) was the variable that most affected the dependent variable. The classification accuracy for this model was 92.3% (*p* = 0.008), meaning that with the divisions established by the model for the independent variables (“BMI” and “Kidmed score”), individuals would be classified in their correct BP category 92/100 times. Accordingly, the percentage of EBP was found to be higher in students with higher BMI values and higher Kidmed scores.

From the third tree diagram results (Figure 4), “body fat percentage” was the independent variable that best explained BP categorization (*p* < 0.001). The classification accuracy for this model was 92.3% (*p* = 0.008), meaning that with the divisions established by the model for the independent variable (“body fat percentage”), individuals would be classified in their correct BP category 92/100 times. Accordingly, the percentage of EBP was higher in students with higher body fat percentages.

## 4. Discussion

This study shows that the prevalence of HT in children aged 6 to 9 years in the province of Valencia was 7.9% and 7.2% in boys and girls, respectively. In addition, the prevalence of PHT in the total sample was 6.1% and 7.5%, respectively. If both elevated BP categories are grouped together, the prevalence of elevated BP is 14.4% for the sample, 14% in boys, and 14.7% in girls. The results confirm a prevalence of normotensive schoolchildren (85.6%), and if we separate by sex, we found that boys had higher rates of HT (7.9%) and girls had higher rates of PHT (7.5%).

A significant global temporal trend of increasing prevalence of EBP among children has been observed over the past 25 years. From 2000 to 2015, the relative increasing rate of HT was 75% to 79% [9]. A 2019 study calculated that the pooled global prevalence of HT among 6-year-old children in 2015 was 4.32% (95% CI, 2.79–6.63%), among 14-year-olds it was 7.89% (95% CI, 5.75–10.75%), the highest in the age range studied, and among 19-year-olds it was 3.28% (95% CI, 2.25–4.77%) [9]. When comparing prevalence rates according to body weight through a subgroup meta-analysis, HT prevalence was higher among overweight (15.27%) and obese (4.99%) children than in normal-weight children (1.90%) [9].

In 2017, the National High Blood Pressure Education Program Working Group on High Blood Pressure in Children and Adolescent updated its normative pediatric BP table by excluding data for overweight and obese children [22]. This modification lowered the BP percentiles utilized to diagnose pediatric EBP, and therefore the current global prevalence of childhood HT might be higher than previously reported. Considering this increasing prevalence and the negative health consequences of EBP in childhood, the need for urgent, all-encompassing measures to prevent, detect, and manage pediatric EBP is imperative [26,27].

Different studies in the worldwide pediatric population have reported significant variability in the prevalence of HT. Published data shows a prevalence of 23% in Chinese children [28], 19.9% in Brazilian children [29], and 12% in children from the Seychelles [30]. However, other studies show percentages more like the results of this study. In a study carried out in Minnesota and California, the reported prevalence of HT was 6.4% [31]. If we compare the results with studies carried out in Spain, the prevalence of HT is lower (7.5% vs. 18.2%) in Castilla-La Mancha [32]. In a previous study within the ANIVA project, the prevalence of HT was 8% and that of PHT was 8.1% [33]. Possible reasons that may explain this variability in HT prevalence include differences in the BP measurement protocols and categorization, obesity trends [34], and the inclusion of participants of different ethnic backgrounds.

Regarding the indicators that could act as markers for elevated BP, BMI, percentage of body fat, and Kidmed scores were identified as the most discerning. The risk of elevated BP increases according to BMI, body fat percentage, and adherence to the Mediterranean diet.

The relationship between anthropometric indicators and different BP components in children has been established in previous scientific literature. Specifically, BMI and body fat percentage have been shown to be moderately and positively correlated with the different BP components [35,36,37], reinforcing that children with higher levels of body fat are more likely to have an increased risk of elevated BP, regardless of the BP component used in their assessment.

Abdominal fat may affect morbidity and mortality in obese individuals, including children [38,39]. It is a major factor in the development of CVD, including elevated BP, in adolescents and adults [40]. Therefore, some of the negative health consequences associated with obesity, which include elevated BP, could affect obese children from an early age. Considering that HT is a serious, underdiagnosed, and poorly controlled problem [41,42], various modifiable factors related to nutrition and lifestyle can play an important role in its control from childhood. This further emphasizes the need to screen children for early detection of overweight/obesity and elevated BP in order to be able to intervene early to modify behaviors and lower CV risk.

### Strengths and Limitations

This study has both limitations and strengths that warrant mentioning. Among the strengths are the sample size, which is representative of the population and large enough for the analysis intended in this study and should not be a source of error. The research question clearly defines the population and outcomes to be investigated; the study design is appropriate and was followed throughout the course of the study. The uniformity of the data collection in both the administration of the questionnaires and the taking of the anthropometric measurements should also be considered a strength of this work. In terms of limitations, the measurement of BP at only one time may be a source of bias, as it is recognized that a lower prevalence of EBP is observed when BP is measured during repeated visits, and there may also be a certain degree of “white coat syndrome” or excitement among the children due to them being accustomed to having their BP measured. Also, there may have been some participation bias as parents were aware of all the data that was to be collected, and those who expected to have “worse” results may have elected not to participate.

## 5. Conclusions

In conclusion, the results obtained in this study show a significant percentage of young schoolchildren with elevated BP in the province of Valencia. Boys had higher rates of HT and girls of PHT. BP values increase as a function of BMI, body fat percentage, and Kidmed scores, making these indicators associated with elevated BP that may be used for screening and early detection.

It is important to identify these at-risk children as early as possible in order to establish appropriate interventions that establish healthy cardioprotective behaviors that must persist through adolescence and into adulthood to prevent the development of CVD. Given the difficulty of individual behavioral change, there is a need for stronger public health programs targeting this specific population. The sole focus cannot be on the identification of children with elevated CVR and public health strategies for maintaining ideal CV health in all children are needed.

## Figures and Tables

**Table 1:** Anthropometric and cardiovascular characteristics.

	Total (*N* = 1142)	Boys (*n* = 543)	Girls (*n* = 599)	*p* Value *
**Age (years)**	7.21 ± 1.00	7.24 ± 0.98	7.19 ± 1.02	0.422
**Height (cm)**	1.39 ± 3.75	1.29 ± 0.15	1.48 ± 5.17	0.391
**Height for age Z-score**	0.43 ± 1.03	0.51 ± 0.98	0.36 ± 108	**0.012**
**Weight (kg)**	28.51 ± 7.08	28.96 ± 7.14	28.10 ± 7.01	**0.039**
**Weight for age Z-score**	0.74 ± 1.75	0.87 ± 2.17	0.62 ± 1.25	**0.018**
**BMI (kg/m^2^)**	17.14 ± 2.82	17.21 ± 2.89	17.08 ± 2.75	0.426
**BMI**				0.062
Underweight	108 (9.5%)	42 (7.7%)	66 (11.0%)	0.057
Normal weight	796 (69.7%)	390 (71.8%)	406 (67.8%)	0.142
Overweight	194 (17.0%)	85 (15.7%)	109 (18.2%)	0.262
Obesity	44 (3.9%)	26 (4.8%)	18 (3.0%)	0.115
**BMI for age Z-score**	0.59 ± 1.23	0.67 ± 1.32	0.51 ± 1.13	**0.028**
**% lean body mass**	34.12 ± 51.39	32.65 ± 48.71	35.46 ± 53.70	0.357
**Waist circunference (cm)**	60.16 ± 15.22	60.46 ± 7.12	59.89 ± 19.89	0.525
**Hip circunference (cm)**	68.13 ± 7.47	68.39 ± 7.64	67.89 ± 7.31	0.261
**Waist-hip index (cm/cm)**	0.96 ± 2.61	1.05 ± 3.78	0.88 ± 0.32	0.280
**Triceps skinfold (mm)**	23.61 ± 38.89	23.54 ± 39.86	23.67 ± 38.02	0.955
**Biceps skinfold (mm)**	14.35 ± 24.44	14.28 ± 25.45	14.42 ± 23.50	0.922
**Abdominal skinfold (mm)**	22.81 ± 39.82	22.59 ± 41.41	23.01 ± 38.35	0.858
**Suprailiac skinfold (mm)**	19.52 ± 40.81	19.84 ± 44.24	19.23 ± 37.47	0.801
**SBP (mm Hg)**	99.76 ± 13.02	100.57 ± 13.07	99.02 ± 12.94	**0.044**
**DBP (mmHg)**	67.78 ± 29.57	67.66 ± 32.17	67.89 ± 27.03	0.897
**Blood pressure category**				0.580
Normal	978 (85.6%)	468 (86.0%)	510 (85.3%)	0.736
Prehypertension	78 (6.8%)	33 (6.1%)	45 (7.5%)	0.349
Hypertension	86 (7.5%)	43 (7.9%)	43 (7.2%)	0.655
**Heart rate (ppm)**	87.76 ± 14.44	86.13 ± 13.92	89.23 ± 1474	**0.001**
MBP (mmHg) **	78.33 ± 21.23	78.52 ± 22.95	78.16 ± 19.55	0.775
PP (mmHg) ***	31.97 ± 29.41	32.91 ± 31.76	31.13 ± 27.09	0.308
**KIDMED score**	7.00 ± 2.04	7.02 ± 2.05	6.97 ± 2.04	0.674
**KIDMED classification**				0.661
Optimal	464 (40.6%)	228 (42.0%)	236 (39.4%)	0.372
Needs improvement	632 (55.3%)	293 (54.0%)	339 (56.6%)	0.378
Bad	46 (4.0%)	22 (4.1%)	24 (4.0%)	0.932

Abbreviations: BMI: body mass index. DBP: diastolic blood pressure. MBP: mean blood pressure. PP: pulse pressure. SBP: systolic blood pressure. Data is presented as mean ± standard deviation or frequency (%). * *p* value (Boys vs. Girls) was calculated using the ANOVA test. Results are considered significant if *p* value ≤ 0.05 and written in bold. ** MBP was calculated as: DBP + [0.33 × (SBP − DBP)]. *** PP was calculated as: SBP − DBP.

## Data Availability

The data presented in this study are available on request from the corresponding author. The data are not publicly available due to privacy and ethical restrictions.

## Supplementary Materials

The following supporting information can be downloaded at: https://www.mdpi.com/article/10.3390/children10121928/s1, Table S1: BMI values used for categorization.
