# Supplementary material for: Cardiovascular Risk Assessment of Elevated Blood Pressure for Screening and Early Detection in Children 6 to 9 Years of Age in the Valencian Community: ANIVA Study
Source: Children (Basel). 2023 Dec 14;10(12):1928. doi: 10.3390/children10121928 (PMC10741757; doi:10.3390/children10121928)
Supplement: Supplementary file 1 [file children-10-01928-s001.zip › children-2750045-supplementary.pdf]

Supplementary Table S1. BMI values used for categorization.

Sex	Age	BMI			
		**Underweight**	**Normal weight**	**Overweight**	**Obese**
**Male**	**6**	≤ 14.07	14.08 - 17.54	17.55 - 19.77	≥ 19.78
	**7**	≤ 14.04	14.05 - 17.91	17.92 - 20.62	≥ 20.63
	**8**	≤ 14.15	14.16 - 18.43	18.44 - 21.59	≥ 21.60
	**9**	≤ 14.35	14.36 - 19.09	19.10 - 22.76	≥ 22.77
**Female**	**6**	≤ 13.82	13.83 - 17.32	17.34 - 19.64	≥ 19.65
	**7**	≤ 13.86	13.87 - 17.74	17.75 - 20.50	≥ 20.50
	**8**	≤ 14.02	14.03 - 18.34	18.35 - 21.56	≥ 21.57
	**9**	≤ 14.28	14.29 - 19.06	19.07 - 22.80	≥ 22.81
